# Volumetric reduction in large cystic jaw lesions postoperative enucleation: a longitudinal clinical study

**DOI:** 10.1186/s12903-023-03307-1

**Published:** 2023-09-13

**Authors:** Sarah Al-Qurmoti, Mueataz A. Mahyoub, Mohamed Elhoumed, Essam Ahmed Al-Moraissi, Zhuo‑Ying Tao, Xiaoru Hou, Jing Li, Sisi Bi, Haiyan Wu, Jing Zhang, Huanhuan Lv, Lina Jiao, Sokina Al-Karmati, Kiran Acharya, Xiaoyi Hu, Jinfeng Li

**Affiliations:** 1 Key laboratory of Shaanxi Province for Craniofacial Precision Medicine Research, College of Stomatology, Xi’an Jiaotong University, 98West 5th Road, Xi’an, 710004 Shaanxi China; 2 Department of Cleft Palate-Craniofacial Surgery, College of Stomatology, Xi’an Jiaotong University, 98West 5th Road, Xi’an, Shaanxi 710004 China; 3 Department of Internal Medicine, The First Affiliated Hospital of Xi’an Jiaotong University, Xi’an, China; 4 Department of Epidemiology and Biostatistics, School of Public Health, Xi’an Jiaotong University Health Science Center, Xi’an, Shaanxi 710061 P.R. China; 5 National Institute of Public Health Research (INRSP), BP. 695, Nouakchott, Mauritania; 6 Department of Oral and Maxillofacial surgery, Thamar university, Dhamar, Yemen; 7 Division of Oral and Maxillofacial Surgery, Faculty of Dentistry, The University of Hong Kong. Prince Philip Dental Hospital, 34 Hospital Road, Sai Ying Pun, Hong Kong; 8 Department of Cranio-Maxillofacial Trauma and Plastic Surgery, College of Stomatology, Xi’an Jiaotong University, Xi’an, China; 9 Department of Stomatology, Shaanxi Provincial Hospital, Xi’an, Shaanxi 710038 China; 10 Department of Craniofacial Surgery, Xi’an Jiaotong University College of Stomatology, 98 West 5th Road, Xi’an, Shaanxi 710004 China; 11 College of Dentistry, Ibb University, Ibb, Yemen; 12 Department of Oral and Maxillofacial Surgery, Xi’an Jiaotong University College of Stomatology, 98 West 5th Road, Xi’an, Shaanxi 710004 China

**Keywords:** Large jaw cysts, Ameloblastomas, Keratocysts, Enucleation, Cavity volume residual, Image J

## Abstract

**Background:**

Enucleation, a surgical procedure, is commonly used to treat large jaw cysts, unicystic ameloblastomas and keratocysts. However, it remains unclear to what extent the jaw bone regenerates after enucleation. We aimed to evaluate the percentage and the survival analysis of jaw bone regeneration, in terms of cavity volume residual (CVR), in patients who underwent enucleation of large jaw cysts, unicystic ameloblastomas and keratocysts.

**Methods:**

We collected data longitudinally from 75 patients who underwent jaw cystic lesions enucleation at the Stomatological Hospital of Xi’an Jiaotong University, between January 2015 and June 2021. All patients had both preoperative and postoperative cone-beam computed tomography (CBCT) imaging data. CBCT images were analyzed using Image J. Changes in the CVR were assessed at various follow-up time points, and the Kaplan-Meier method was utilized to evaluate the CVR over time.

**Results:**

The patients had a mean age of 31.7 years (range: 5.5–72 years) with 58.66% of them being male. The postoperative CVR was 32.20% at three months, 21.10% at six months, 15.90% at 12 months, and 5.60% at 24 months. The percentage of CVR during follow-up periods for the initial size Quartile (Q)1 (212.54-1569.60 mm^3^) was substantially lower than those of Q2 and Q3 at and after seven months of follow-up and became statistically significant at the 12-month mark.

**Conclusion:**

This study demonstrates that spontaneous bone regeneration can occur after enucleation of large jaw cysts, unicystic ameloblastomas and keratocysts, even without the use of filler materials. The initial size of the lesion had a significant impact on the outcome of cystic lesion enucleation over time. To minimize the risks associated with radiation exposure and expenses, we recommend reducing the frequency of CT imaging follow-ups for patients with small initial cavity sizes (ranging from 212.54 to 1569.60 mm^3^).

## Introduction

Odontogenic jaw cysts can develop at any point in life and have a prevalence rate of 2.4–6.4% in the general population. The location of these cysts in the upper or lower jaw depends on their origin [1, 2]. These cysts typically have one or more cavities lined by a specialized cyst epithelium [3]. Most jaw cysts are well-defined, oval-shaped radiolucent lesions that vary in size and origin [4]. Enucleation of the cyst is a widely accepted treatment modality that involves removing the cystic wall to prevent recurrence [5, 6]. Other commonly used techniques include marsupialization and decompression, which aim to create bone by reducing the pressure on the lesion [7]. The success of these techniques depends on the size of the cyst defect and the duration of observation after surgery, which can affect postoperative bone healing [8, 9]. The prognosis for small cysts can be favorable with little morbidity, depending on the size of the cyst. For example, a study found that 97% of bone density regenerated 12 months after surgery for small 2–3 cm defects [10]. A systematic review by Nyimi et al. [11]. found that cysts less than 4 cm could regenerate to a suitable bone density 24 months after enucleation. However, large cysts have higher postoperative risks, such as pathological fracture, restricted opening, and inadequate bone healing, making their treatment more challenging [12].

The utilization of autogenous grafts or implant substances in the treatment of large cystic cavities has limitations. Contaminated specimens can cause infection in the recipient’s site, resulting in disease transmission from donor to recipient. Additionally, incompatibility with the host, surgical treatment incompetence, the recommendation for a secondary surgical donor site, increased morbidity after surgery, and prolonged surgery time can be problematic [13]. Several studies have been carried out over an extended period to determine the degree of bone regeneration by calculating the maximum diameters of the residual cavities at various times after the cyst’s enucleation [3]. Previous studies have shown that bone healing can occur in all patients with cysts at 6, 12, and 24 months after surgery [14]. Nevertheless, these studies featured a small number of participants and did not encompass cases of unicystic ameloblastomas and keratocysts.

There is still disagreement about spontaneous bone regeneration following cyst enucleation, as several variables such as cyst size prior to surgery, patient age, and gender [15]. Previous studies have used orthopantomogram, a 2-dimensional analysis method, to evaluate volumetric changes in cysts [16]. However, this method has limitations in accurately identifying the volumetric variation. On the other hand, cone-beam computed tomography (CBCT) provides a highly accurate and reproducible 3D volumetric methodology for evaluating bone regeneration after the enucleation of cystic lesions. The extent of jaw bone regeneration after enucleation, especially for large jaw cysts, unicystic ameloblastomas and keratocysts (≥ 2 cm), remains unclear. In this study, we aimed to assess the degree of spontaneous bone regeneration, in terms of cavity volume residual (CVR), in patients who underwent enucleation of large jaw cystic lesions using CBCT scans. We also examined the correlation between the degree of spontaneous bone regeneration, indicated by CVR, and various patient characteristic factors such as age, gender, and cyst size at the beginning, to gain a deeper understanding of the clinical implications of these factors on bone healing. The results of this study may provide valuable information for clinicians to optimize the treatment of patients with odontogenic jaw cysts.

## Methods

### Study design and participants

We conducted a longitudinal study and included 75 patients (44 males and 31 females) who underwent jaw cyst, unicystic ameloblastomas and keratocysts enucleation between January 2015 and June 2021 at the Department of Oral and Maxillofacial Surgery, Stomatological Hospital of Xi’an Jiaotong University.

### Inclusion criteria

Patients were included if they (1) had maxillary or mandibular cystic lesions larger than 2 cm, which underwent enucleation without bone grafting, and had preoperative and postoperative CT scans available for analysis at least six months after surgery; and (2) were histopathologically diagnosed with dentigerous cysts (DC), keratocysts (KC), periapical cysts (PC), paradental cysts (PDC), or unicystic ameloblastomas (AM). Patients were excluded if they (1) had confirmed nevoid basal cell carcinoma syndrome; or (2) had multiple or recurrent lesions; or (3) were treated using artificial or natural filler materials.

### Data collection

We collected clinical information, including age, gender, lesion location, lesion size, length of follow-up, and histopathological diagnosis. We measured the volume of the cystic cavities using preoperative and postoperative CBCT scans (DCT PRO CBCT, Vatech, Co., Ltd., Hwasung, Korea) with the following regimen: 7 mA, 90 kV, 512 × 512 matrix, field of view of 20 × 19 cm, 24-second scanning time and 0.4 voxel scanning resolution.

The CVR was defined as the difference between the preoperative cavity volume (T0) and the postoperative cavity volume at a specific follow-up time (Tx), where x denotes the duration since the surgical intervention.

### Measurement of the preoperative and postoperative cavity volume

**Fig. 1:**
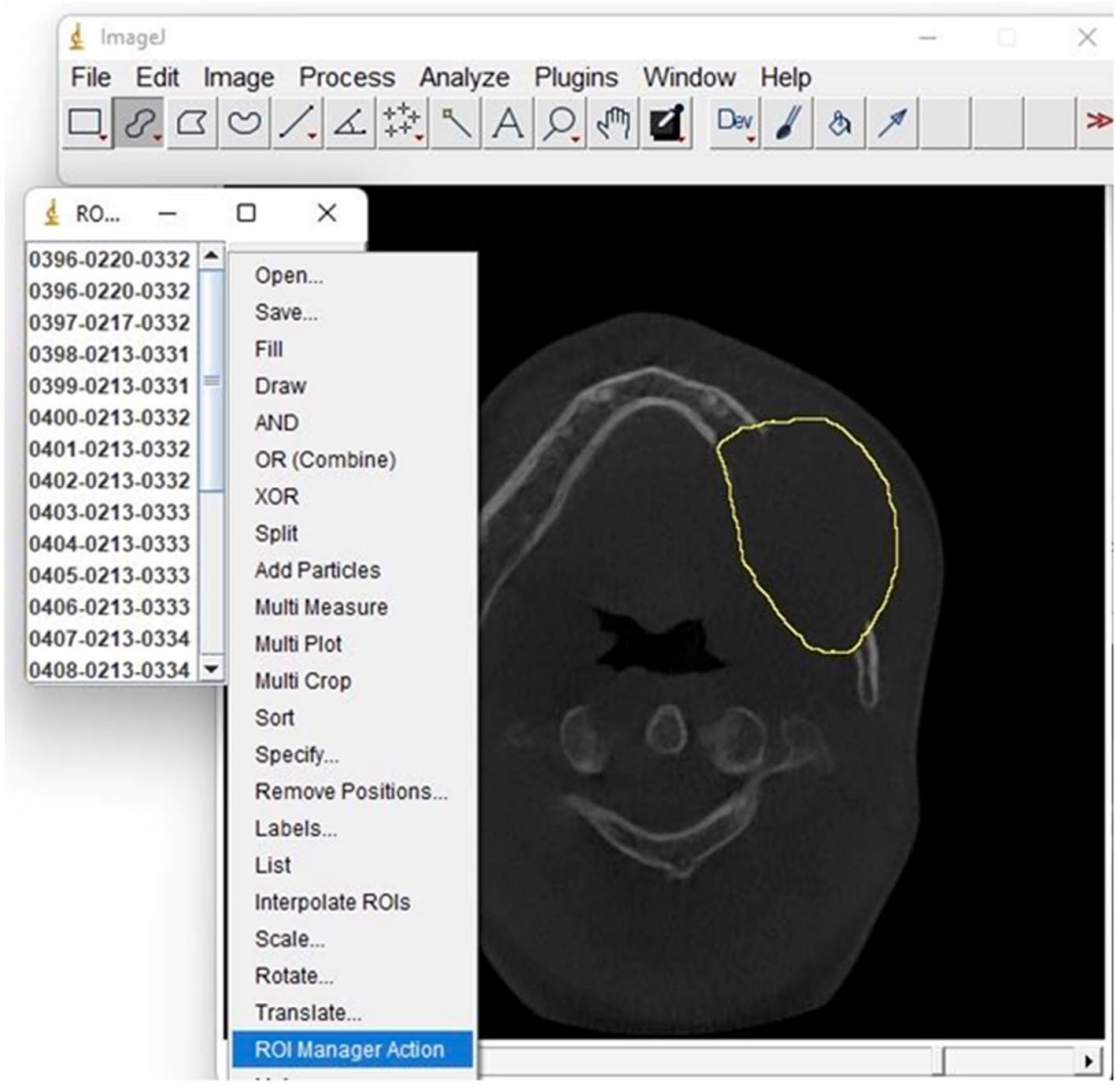
Using the ImageJ software for measuring the volume of a jaw cystic lesion before and after enucleation

The CBCT images were first opened in Image J (v. 1.51k) in an appropriate 8-bit grayscale format. The region of interest (ROI), which includes the cyst, was then defined using Image J’s selection tools. The cyst was separated from the surrounding tissue using Image J’s segmentation tools, such as thresholding or watershed. The dimensions of the cyst in 3D, including its volume, surface area, or diameter, were then measured using ImageJ’s measurement tools by drawing a line along the cyst and residual cavity on the axial section of a CBCT image using the freehand selection tool. The properties of the interpolated ROI, such as its length, area, or shape, were also measured using Image J’s measurement tools. In order to generate a more precise measurement, the interpolation tool in ImageJ was used to smooth out the cyst’s shape, and the procedures were carried out by two dentists (S.Q.) and (S.K.). The measurement results were then recorded and exported to Excel for further analysis. By following this methodology, the size of the preoperative and postoperative cavity volumes was accurately measured and recorded for further analysis in this study (Fig. 1).

### Statistical analyses

Stata version 17 (Texas, USA) was used to calculate all the statistical analyses. Participants’ characteristics were presented as numbers and percentages for categorical variables and mean (max, min) for continuous variables. Then, CVR changes were presented during the follow-up time points.

Meanwhile, our data were transformed into a longitudinal form (panel data) using the Stata command “*reshape*” [17]. The Kaplan-Meir curve method was applied for further analysis of the CVR evaluation during each follow-up time point. However, our data is snapshot data (individuals differ at each time point). We did data transformation to time-span data using the Stata command “*snapspan”* using CVR “*event*” as the outcome that occurs at the instant of the snapshot that is to apply to the time span ending at the “time” of the current snapshot.

The Kaplan-Meir curve of the CVR evaluation was presented for the overall stratified by gender, age, and initial size of the cystic lesion during follow-ups from 0 to 60 months.

## Results

### Distribution of participants according to parameters

The patients’ mean age was 31.7 years (range,5.5 to 72 years), and forty-four (58.66%) of patients were male. Of the 75 jaw cystic lesions, 28 (37.33%) were located in the maxilla and 47 (62.67%) in the mandible. The patients were diagnosed with a variety of cystic lesion types, including paradental cysts (*n* = 32, 43%), dentigerous cysts (*n* = 6, 6.75%), keratocysts (*n* = 7, 9.33%), periapical cysts (*n* = 18, 24%), and unicystic ameloblastoma (*n* = 12, 16%), as shown in Table 1.

**Table 1:** General characteristics of participants

Parameters		Parameters number	Percentage
Gender	Male Female	44 31	58.66% 41.33%
Age	5.5 ~ 21 22 ~ 42 43 ~ 72	25 30 20	33.33% 40.00% 26.66%
Location	Maxilla Mandible	28 47	37.33% 62.67%
Pathological classification	Dentigerous cyst Periapical cyst Keratocysts Paradental cyst Ameloblastoma	6 18 7 32 12	8.00% 24.00% 9.33% 42.67% 16.00%

The mean initial cavity volume of the participants was 6184.29 mm^3^, the maximum was 56175.00 mm^3^, and the minimum was 212.54 mm^3^. Three months after jaw cystic lesions enucleation, the mean cavity volume was 2523.71 mm^3^, the maximum was 11833.98 mm^3^, and the minimum was 24.45 mm^3^. At six months, the mean cavity volume was 1880.59 mm^3^, the maximum was 6068.03 mm^3^, and the minimum was 33.41 mm^3^. At 12 months, the mean cavity volume was 688.02 mm^3^; the maximum was 4662.70 mm^3^, and the minimum was 0.00 mm^3^. Finally, 24 months after jaw cystic lesions enucleation, the mean cavity volume was 340.38 mm^3^, the maximum was 1301.12 mm^3^, and the minimum was 0.00 mm^3^, as shown in Table 2. All participants had no chronic diseases and were nonsmokers.

**Table 2:** Pre and postoperative cavity volume of participants

Cavity volume (mm^3^)	Mean	Minimum	Maximum
T0 (Before)	6184.29	212.54	56175.00
T1 (3 Months)	2523.71	24.45	11833.98
T2 (6 Months)	1880.59	33.41	6068.03
T3 (12 Months)	688.02	0.00	4662.70
T4 (24 Months)	340.38	0.00	1301.12

T0, Before jaw cystic lesions enucleation; T1 Cavity volume at three months after enucleation; T2, Cavity volume at six months after enucleation; T3 Cavity volume at 12 months after enucleation; T4 Cavity volume at 24 months after enucleation

### Changes in the cavity volume residual

**Fig. 2:**
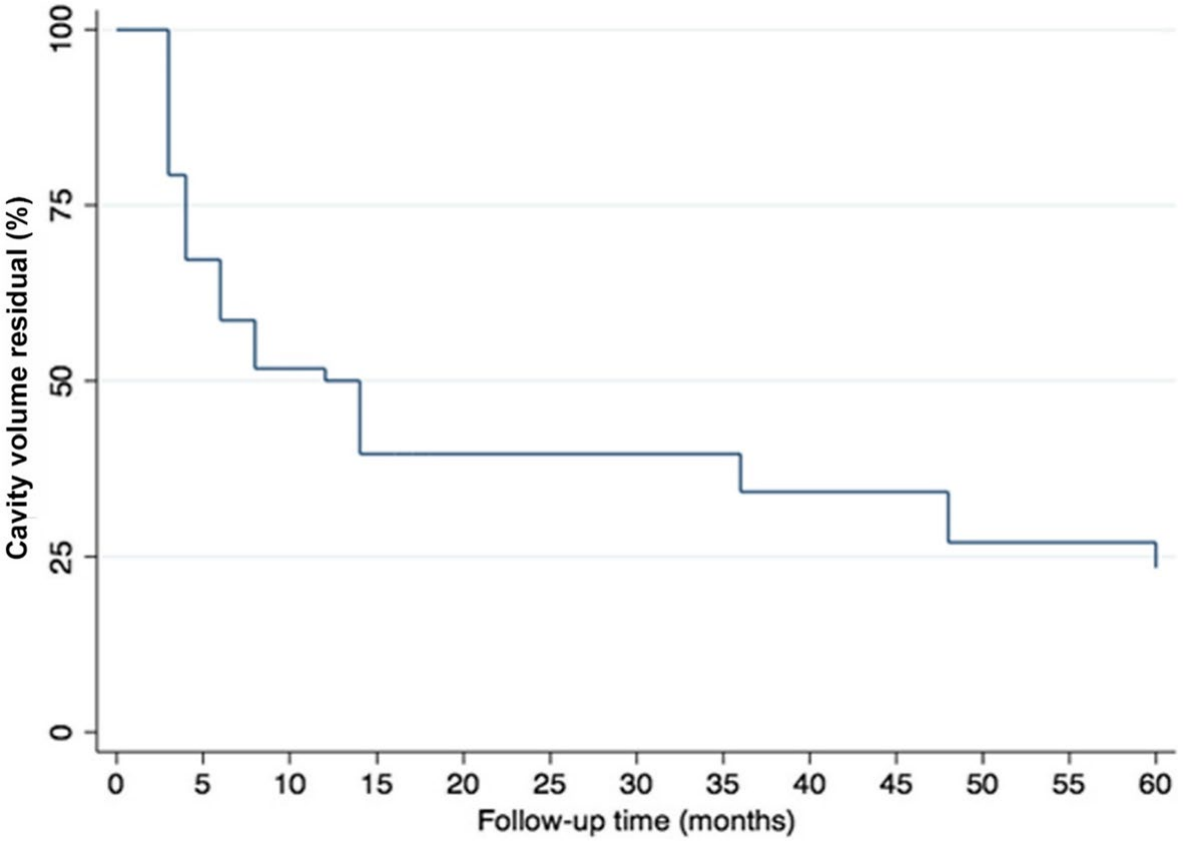
The overall Kaplan-Meier Curve analysis of the percentage cavity volume residual (CVR) during follow-up periods following jaw cystic lesions enucleation

The overall percentage of postoperative CVR was (32.20) % at three months, (21.10) % at six months, (15.90) % at 12 months, and (5.60) % at 24 months. The percentage of CVR was gradually reduced after jaw cystic lesions enucleation, as shown in Table 3. The period required following jaw cystic lesions enucleation for the decrease in CVR percentages was marked by fast from 0 to 6 months and slow and steady from 6 to 60 months (see Fig. 2).

**Table 3:** Changes in the cavity volume residual during the follow-ups

Difference	Follow up	*n*	CVR (mm^3^)	Minimum (mm^3^)	Maximum (mm^3^)	CVR Percentage
T1-T0	3 Months	14	4079.48	61.12	27845.44	32.20
T2-T0	6 Months	11	5683.82	1587.68	37010.24	21.10
T3-T0	12 Months	13	2779.01	474.40	30081.92	15.90
T4-T0	24 Months	16	7008.56	460.32	45088.47	5.60

*CVR *Cavity volume residual, *T0 *Jaw cystic lesions volume on the day of enucleation, *T1 *Cavity volume at three months after enucleation, *T2 *Cavity volume at six months after enucleation, *T3 *Cavity volume at 12 months after enucleation, *T4 *Cavity volume at 24 months after enucleation

The CVR (the difference between the preoperative cavity volume (T0) and the postoperative cavity volume at a specific follow-up time (Tx)), where x denotes the duration since the surgical intervention

### Analysis of factors influencing CVR

#### Longitudinal estimation of the percentage CVR during follow-up periods following jaw cystic lesions enucleation by gender

**Fig. 3:**
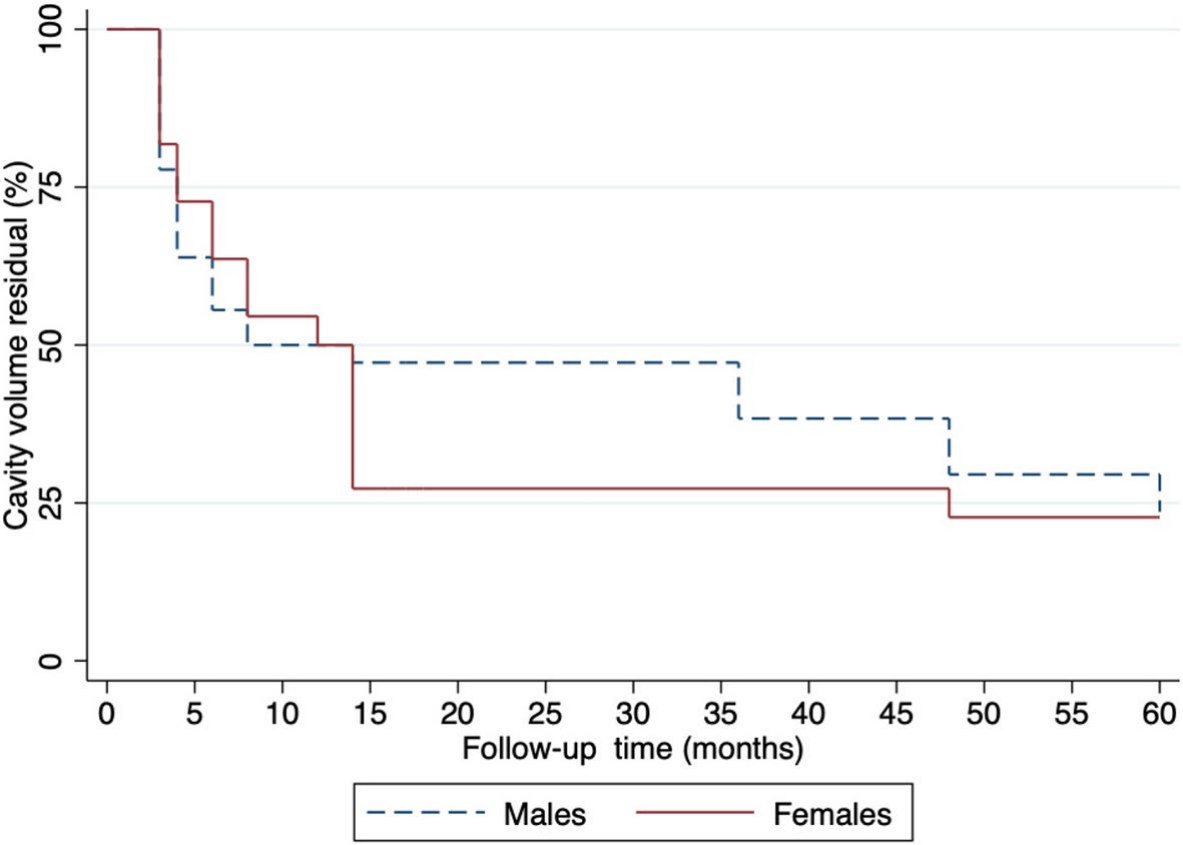
The Kaplan-Meier Curve analysis of the percentage cavity volume residual (CVR) during follow-up periods following jaw cystic lesions enucleation stratified by gender

The Kaplan–Meier curve analysis shows that, overall, the percentage of CVR during follow-ups periods of male patients was less compared to female patients before 15 months and while the percentage of CVR during follow-ups periods of female patients was less compared to male patients after 15 months (see Fig. 3).

#### Longitudinal estimation of the percentage CVR during follow-up periods following jaw cystic lesions enucleation by age

**Fig. 4:**
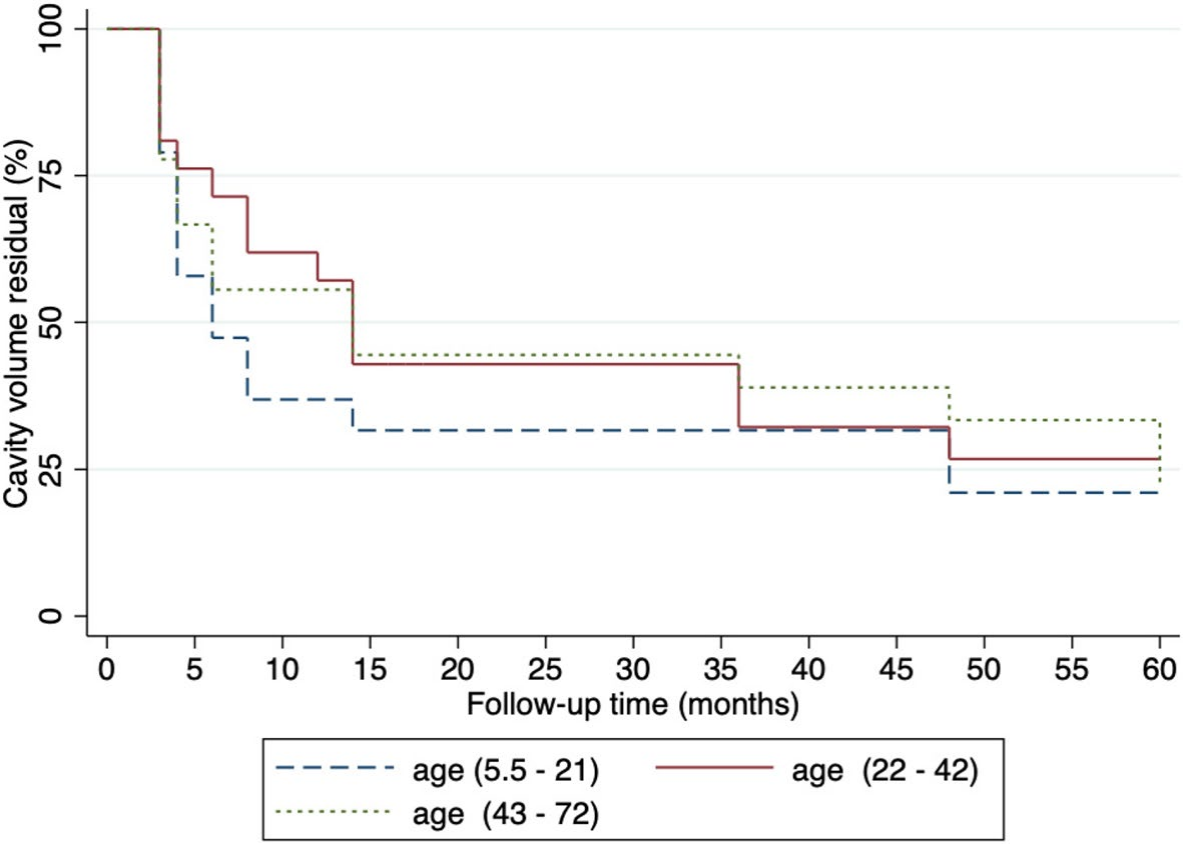
The Kaplan-Meier Curve analysis of the percentage cavity volume residual (CVR) during follow-up periods following jaw cystic lesions enucleation stratified by Age groups

In the present study, we investigated the percentage of CVR during follow-up periods across different age groups. The results obtained from the Kaplan-Meier curve analysis revealed a substantial difference in the percentage of CVR between the age group of 5.5 to 21 years and other age groups (see Fig. 4). Specifically, the percentage of CVR was markedly lower in the age group (5.5 to 21 years), indicating a more effective jaw cystic lesions enucleation in this age range. These findings suggest that age may play a crucial role in the treatment outcome and should be considered in the clinical management of patients undergoing jaw cystic lesions enucleation.

#### Impact of initial lesion size on the percentage of CVR following enucleation

**Fig. 5:**
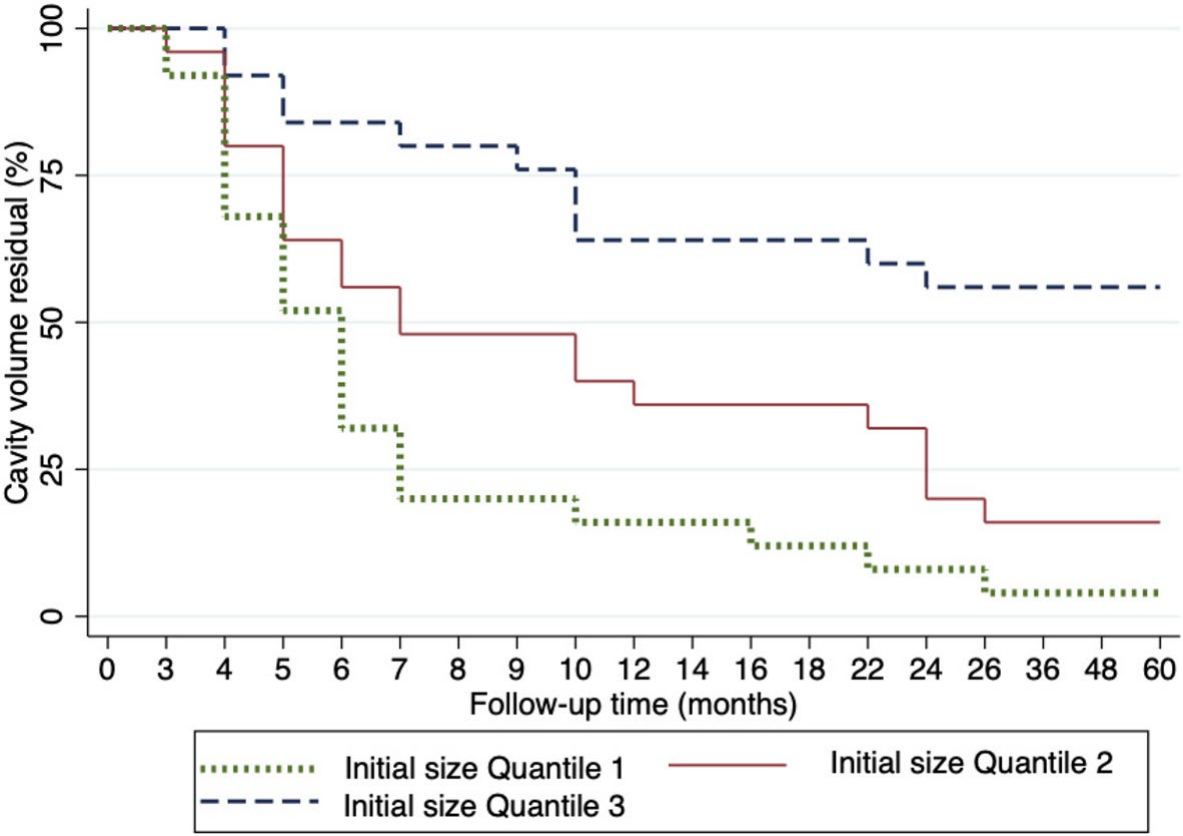
Kaplan-Meier Curve analysis of the percentage of cavity volume residual (CVR) during follow-up periods following jaw cystic lesions enucleation, stratified by initial size quantiles. The initial size quantiles include Q1 (212.54-1569.60 mm^3^), Q2 (1605.31-6524.22 mm^3^), and Q3 (6618.11-56175.55 mm^3^)

Based on the findings of the Kaplan-Meier curve analysis, it can be concluded that the percentage of CVR during the follow-up periods of the initial size Quartile (Q) 1 (212.54-1569.60 mm^3^) was markedly lower compared to those of Q2 and Q3 (see Fig. 5). This difference was particularly evident at and after seven months of follow-up and became statistically significant at the 12-month mark. Therefore, these results suggest that the lesion’s initial size may significantly impact the outcome of jaw cystic lesions enucleation over time.

## Discussion

Regeneration of the jaw bone without filler material is generally accepted for small cysts, but the use of filler material for the treatment of large cysts, unicystic ameloblastomas and keratocysts is still a topic of debate among the researchers and practitioners. Some studies suggest that the use of filler material can help with the healing process, while others advocate for spontaneous bone regeneration without filler materials [18]. This disagreement highlights the lack of consensus.

This study aimed to assess the percentage and the survival curve of jaw bone regeneration without filler material in terms of cavity volume residual (CVR) in patients undergoing the removal of large jaw cysts, unicystic ameloblastomas and keratocysts. Using a three-dimensional CBCT scan and Image J software, we measured the CVR percentages of large jaw cysts, unicystic ameloblastomas and keratocysts without filler materials. Our results indicated a significant decrease in CVR percentages of large jaw cysts, unicystic ameloblastomas and keratocysts after enucleation without filler materials compared to the day of operation, which is consistent with previous research [13, 19, 20]. However, a prospective randomized study found no significant difference in bone generation rate at three months after enucleation between the groups with and without filler materials. Additionally, the study found a significant difference at six months after enucleation, with a higher rate of bone generation observed in the group that used filler materials [21]. These findings do not coincide with our results, and they could be due to differences in methodology, study design, demographics, or insufficient management of confounding factors.

In our study, we observed a notable decline in CVR percentages after jaw cystic lesions enucleation. The decline was rapid from 0 to 6 months, followed by a slow and steady decrease from 6 to 60 months. This finding is in contrast to a previous study that reported a decline in CVR percentages from 3 to 12 months [22]. We also discovered that female patients had a slightly lower CVR percentage than male patients before 15 months. However, after 15 months, the CVR percentage was significantly lower in female patients than in male patients. Previous research has shown that females may have slower bone healing and a higher risk of nonunion, which is the failure of the bone to heal [23, 24]. Hormonal differences may play a role, as estrogen has been found to inhibit bone formation and promote bone resorption. Additionally, females tend to have smaller jaw bones, which could also impact the healing process. However, conflicting results have been reported in other studies, with no significant differences observed in bone healing and regeneration between males and females [20]. The underlying reasons for these discrepancies are not well understood, and further research is needed to gain a better understanding of the impact of gender on bone healing.

Furthermore, our study found that the percentage of CVR during the follow-up period was significantly lower in the age group of 5.5 to 21 years compared to other age groups. This result is consistent with previous studies [25, 26] found that the CVR percentage in young patients to be lower than that in older patients. However, another study reported that patient age does not affect CVR [11], which contradicts our findings. The reason for our results may be due to age-related changes in bone healing and regeneration. In older adults, bone healing and regeneration are slower due to factors such as decreased blood flow to the healing site, decreased numbers and function of osteoblasts, and increased numbers and activity of osteoclasts. Additionally, age-related changes in the immune system may also impact the healing process. Moreover, changes in the levels of growth factors and signaling molecules can also affect bone regeneration, as studies have shown that levels of bone morphogenetic proteins and other growth factors decrease with age, leading to decreased bone formation and slower healing [27, 28]. In contrast, children and adolescents experience active growth, leading to faster and more active bone formation and remodeling, resulting in faster bone regeneration after enucleation [29].

In our study, we observed that the percentage of CVR during the follow-up period was significantly lower in lesions with small initial sizes than those with large initial sizes. This difference was most noticeable at and after seven months of follow-up and became statistically significant at 12 months. Previous research has indicated that spontaneous bone regeneration, as indicated by CVR, is greater in cases where the cystic lesion’s initial size is larger [9, 29]. In contrast, Anavi et al. found that the relative rate of decrease was higher in cases with smaller initial sizes, which is consistent with our findings [26]. However, it is essential to note that the interpretation of their results may be complex since the rate of absolute CVR decrease could yield different outcomes.

There were limitations to our study, as it was conducted retrospectively, and therefore we were unable to analyze all factors that may contribute to CVR time, including chronic diseases, alcohol consumption, and smoking index. Additionally, because it was a hospital-based study conducted at a single hospital, it is possible that differing referral patterns may have caused selection bias. We are confident that there was no ascertainment bias as all patients had clinically and histopathologically proven cysts. The diagnostic confirmation of cystic lesions and the risk factor data were of the highest quality, as comprehensive demographic and clinical data were carefully obtained for every patient at their initial examination at Stomatology Hospital of Xi’an Jiaotong University. However, it is worth noting that our sample size was relatively small, and we used longitudinal analyses with multiple time points of follow-up.

## Conclusion

Our study findings indicate that the omission of filler materials did not affect the volumetric reduction of large jaw cysts, unicystic ameloblastomas and keratocysts after enucleation. The decline in the rate of CVR is influenced by various factors, including the size of the jaw cystic lesions at the initial stage, patient age, and gender. The decline is initially rapid during the first six months, followed by a gradual decrease from six to 60 months. To minimize the risks associated with radiation exposure and expenses, we recommend reducing the frequency of CT imaging follow-ups for patients with small initial cavity sizes (ranging from 212.54 to 1569.60 mm^3^). However, further research involving larger sample sizes is necessary to validate these findings and investigate bone regeneration after the enucleation of large jaw cysts, unicystic ameloblastomas and keratocysts.

## Data Availability

The datasets used and/or analyzed during the study are available from the corresponding author on reasonable request.

## Abbreviations

CBCT: Cone beam computed tomography
CVR: Cavity volume residual
DC: Dentigerous cyst
KC: Keratocyst
PC: Periapical cyst
PDC: Paradental cyst
AM: Ameloblastoma
